# Platelet Function Disorders: Glanzmann Thrombasthenia and Type 2 Von Willebrand Disease

**DOI:** 10.7759/cureus.111287

**Published:** 2026-06-22

**Authors:** Navin Raj Ramamurthy, Shashank M, Sugil E, Selvakumar Selvaraj, Namitha Narayanan, Ashokraj Selvam, Dhivyapriya S

**Affiliations:** 1 Institute of Internal Medicine, Madras Medical College, Chennai, IND; 2 Internal Medicine, Madras Medical College, Chennai, IND

**Keywords:** adolescent bleeding disorders, bleeding disorder, glanzmann thrombasthenia, mucocutaneous bleeding, platelet function disorders, type 2 von willebrand disease

## Abstract

Bleeding disorders represent a broad spectrum of conditions that disrupt normal clot formation through abnormalities in platelet function or the coagulation cascade. Disorders affecting primary hemostasis typically manifest as mucosal bleeding, whereas abnormalities in secondary hemostasis more often lead to deep tissue hemorrhage. However, certain conditions, such as von Willebrand disease, may involve features of both pathways, complicating clinical evaluation. This report describes two adolescent patients who presented with mucocutaneous bleeding but were ultimately diagnosed with different underlying conditions: Glanzmann thrombasthenia and type 2 von Willebrand disease. The first patient exhibited recurrent gingival bleeding with normal platelet counts and near-normal coagulation studies. Further evaluation using platelet aggregation testing revealed impaired responses to multiple agonists, consistent with a defect in platelet aggregation. The second patient presented with gingival bleeding and severe menorrhagia and was initially treated as a case of hemophilia due to reduced factor VIII levels. Lack of therapeutic response prompted further testing, which demonstrated abnormal von Willebrand factor activity, confirming a qualitative defect. These cases highlight the importance of correlating clinical presentation with specialized laboratory investigations. Reliance on routine tests alone may lead to misdiagnosis, whereas a systematic diagnostic approach allows accurate identification and appropriate management of bleeding disorders.

## Introduction

Disorders of hemostasis encompass a wide variety of conditions that interfere with the body’s ability to control bleeding. They are broadly classified into disorders of primary hemostasis, involving platelet adhesion, activation, and aggregation leading to platelet plug formation, and disorders of secondary hemostasis, involving abnormalities of the coagulation cascade and fibrin formation [1].

Bleeding patterns often provide important diagnostic clues. Superficial bleeding manifestations such as epistaxis, gum bleeding, petechiae, and heavy menstrual bleeding are typically associated with platelet-related disorders. In contrast, bleeding into joints or muscles is more suggestive of coagulation factor deficiencies. However, despite important clues, a distinction between disorders of adhesion, activation, and aggregation is unclear [2]. Although these defects have distinct pathological mechanisms, they commonly present with similar superficial bleeding symptoms, making clinical differentiation challenging. Specialized laboratory investigations are therefore essential to establish the correct diagnosis, as management strategies and long-term outcomes vary among these disorders [2].

Glanzmann thrombasthenia is an inherited platelet function disorder characterized by defective platelet aggregation due to abnormalities in the glycoprotein IIb/IIIa integrin receptor evidenced by lack of in vitro coagulation to all soluble agonists [3]. Von Willebrand disease, the most prevalent inherited bleeding disorder, is inherited equally in men and women and arises from either reduced levels or impaired function of von Willebrand factor. Among its subtypes, type 2 disease is defined by qualitative defects that alter its functional activity [4].

Accurate differentiation between these conditions is essential, as their management strategies differ significantly. This report presents two cases that illustrate the diagnostic challenges encountered in adolescents presenting with mucocutaneous bleeding.

## Case presentation

Case 1: Glanzmann thrombasthenia

Clinical Presentation

A 13-year-old adolescent female presented with a history of recurrent mucocutaneous bleeding, predominantly spontaneous gum bleeding. The onset was insidious, with symptoms developing over a few years since five years of age. The bleeding episodes were recurrent, prolonged, and occurred without any identifiable precipitating factors such as trauma or dental procedures. Over time, the patient experienced progressive pallor, generalized weakness, easy fatigability, and exertional breathlessness.

There was no history of bleeding from other sites such as epistaxis, hematemesis, melena, or hematuria. Importantly, there was no history of deep tissue bleeding, hemarthrosis, or muscle hematomas. There was no history suggestive of systemic illness, drug intake, or toxin exposure. Past medical history was unremarkable, with no prior hospitalizations or transfusions before the current episode. Family history was negative for bleeding disorders.

On examination, she was conscious and hemodynamically stable. She had marked pallor but no icterus, cyanosis, or edema. An additional notable finding was delayed pubertal development. Local examination revealed active gum bleeding. Systemic examination, including cardiovascular, respiratory, abdominal, and neurological systems, was unremarkable.

Workup

Initial laboratory investigations demonstrated severe anemia with a hemoglobin level of 5.4 g/dL. The platelet count was within normal limits, and total leukocyte count was normal, as shown in Table 1. Peripheral smear examination showed dimorphic anemia with both normocytic normochromic and microcytic hypochromic red blood cells, along with anisopoikilocytosis, consistent with chronic blood loss anemia. Platelet morphology was largely normal, excluding structural platelet abnormalities.

**Table 1:** Laboratory data: initial investigations. WBC: white blood cell; RBC: red blood cell; MCV: mean corpuscular volume; MCH: mean corpuscular hemoglobin; MCHC: mean corpuscular hemoglobin concentration

Investigation	Value	Reference range
Hemoglobin	5.4 g/dL	12–15 g/dL
Total WBC count	7,800 cells/mm³	4,500–11,000 cells/mm³
RBC count	2.65 million cells/mm³	3.92–5.4 million cells/mm³
Platelet count	1,68,000 platelets/mm³	150,000–450,000 platelets/mm³
Hematocrit	22.1%	36–48%
MCV	83.4 fL	80–100 fL
MCH	20.4 pg/cell	27–33 pg/cell
MCHC	24.4 g/dL	32–36 g/dL
Neutrophils	47%	40–75%
Lymphocytes	45%	20–45%
Monocytes	6%	2–10%
Eosinophils	2%	0–7%

Coagulation studies revealed near-normal prothrombin time and international normalized ratio (INR), with a mildly prolonged activated partial thromboplastin time. These findings did not indicate a significant coagulation factor deficiency. Given the presence of mucocutaneous bleeding with normal platelet counts and largely preserved coagulation parameters, a qualitative platelet disorder was suspected.

Further evaluation with platelet aggregation studies revealed markedly reduced aggregation responses to ADP, collagen, and arachidonic acid, with preserved aggregation in response to ristocetin (Table 2). This pattern is characteristic of Glanzmann thrombasthenia, reflecting a defect in platelet aggregation due to dysfunction of glycoprotein IIb/IIIa receptors.

**Table 2:** Coagulation workup: laboratory data. PT: prothrombin time; INR: international normalized ratio; aPTT: activated partial thromboplastin time; vWF: von Willebrand factor; ADP: adenosine diphosphate

Investigation	Value	Reference range
PT	11.0 seconds	11–14 seconds
INR	0.99	0.9–1.5
aPTT	24 seconds	24–36 seconds
Thrombin time	17.3 seconds	14–21 seconds
Fibrinogen level	259 mg/dL	200–400 mg/dL
Factor VIII assay (clot-based assay)	246%	50–150%
Factor IX assay (clot-based assay)	102%	50–150%
Factor XIII (chromogenic assay)	91%	50–150%
vWF antigen assay (clot-based assay)	144%	50–150%
vWF ristocetin (light transmission aggregometry assay)	64.7%	50–200%
ADP (light transmission aggregometry assay)	7.0%	50–100%
Arachidonic acid (light transmission aggregometry assay)	0.2%	50–100%
Collagen (light transmission aggregometry assay)	14.7%	50–100%
Ristocetin (light transmission aggregometry assay)	20.9%	50–100%

Endocrine evaluation revealed a bone age of approximately 10 years with low gonadotropin and estradiol levels (Table 3), along with imaging findings of an infantile uterus. These findings were consistent with delayed puberty, for which conservative management was advised.

**Table 3:** Laboratory data: delayed puberty. ACTH: adrecorticotropic hormone; LH: luteinizing hormone

Investigation	Value	Reference range
ACTH	10.7 pg/mL	10–60 pg/mL
LH	<0.1 IU/L	<0.02–4.8 IU/L
Estradiol	<10 pg/mL	<10 pg/mL
Prolactin	7.16 ng/mL	3–20 ng/mL

Additional investigations were performed to exclude secondary causes. Direct Coombs test was negative, ruling out autoimmune hemolysis. Viral markers were negative, and abdominal ultrasonography did not reveal any abnormalities. Karyotyping was found to be of a normal female chromosome.

Diagnosis

Based on the integration of clinical features and laboratory findings, a diagnosis of Glanzmann thrombasthenia, a qualitative platelet aggregation disorder, was established. The hallmark features included mucocutaneous bleeding, normal platelet count, normal coagulation profile, and characteristic abnormalities in platelet aggregation studies. The pathophysiology involves a defect in platelet glycoprotein IIb/IIIa receptors, impairing fibrinogen-mediated platelet aggregation. This results in defective primary hemostasis despite normal platelet numbers.

Management and Outcome

The patient was managed with supportive therapy, including packed red blood cell transfusions for severe anemia and antifibrinolytic therapy with tranexamic acid to control bleeding. She was closely monitored, and bleeding episodes gradually subsided with treatment.

Case 2: Type 2 von Willebrand disease

Clinical Presentation

A 14-year-old adolescent female presented with persistent mucocutaneous bleeding and severe menorrhagia since menarche. The bleeding was characterized by heavy menstrual flow requiring the use of multiple pads per day over prolonged durations and was severe enough to necessitate blood transfusions. The symptoms recurred despite initial treatment, significantly affecting her quality of life.

There was no history of petechiae, purpura, or bleeding following minor trauma. Additionally, there was no history of other deep tissue bleeding, hemarthrosis, or systemic illness. Past medical history was unremarkable, and there was no family history of bleeding disorders.

On examination, the patient was hemodynamically stable with pallor but no other significant findings. There were no cutaneous signs of bleeding, and systemic examination was normal.

Workup

Laboratory investigations revealed moderate anemia. Platelet count was normal to elevated, as shown in Table 4. Peripheral smear examination showed normocytic normochromic anemia without abnormal platelet morphology. Coagulation studies revealed a normal prothrombin time and INR, with a mildly prolonged activated partial thromboplastin time, suggesting an intrinsic pathway abnormality. Factor VIII levels were found to be reduced (approximately 17%), leading to an initial diagnosis of hemophilia A.

**Table 4:** Laboratory data. RBC: red blood cell; WBC: white blood cell; MCV: mean corpuscular volume; MCH: mean corpuscular hemoglobin; MCHC: mean corpuscular hemoglobin concentration; RDW-CV: red cell distribution width-coefficient of variation; AST: aspartate aminotransferase; ALT: alanine aminotransferase

Investigation	Value	Reference range
Hemoglobin	8.8 g/dL	12–15 g/dL
Total WBC count	6,100 cells/mm³	4,500–11,000 cells/mm³
RBC count	3.17 million cells/mm³	4.2–5.4 million cells/mm³
Platelet count	547,000 cells/mm³	150,000–415,000 cells/mm³
Hematocrit	28.8%	36–44%
MCV	90.1 fL	80–100 fL
MCH	27.8 pg/cell	27–33 pg/cell
MCHC	30.7 g/dL	32–36 g/dL
Neutrophils	60%	40–70%
Lymphocytes	30.4%	20–50%
Monocytes	9.4%	2–10%
RDW-CV	14.9%	11.5–14.5%
Urea	18 mg/dL	15–40 mg/dL
Creatinine	0.7 mg/dL	0.6–1.2 mg/dL
Sodium (Na^+^)	132 mEq/L	135–145 mEq/L
Potassium (K^+^)	4.7 mEq/L	3.5–5 mEq/L
Total bilirubin	0.2 mg/dL	0.3–1.2 mg/dL
Direct bilirubin	0.1 mg/dL	<0.3 mg/dL
Total protein	7.1 g/dL	6–8 g/dL
Albumin	4.3 g/dL	3.5–5 g/dL
AST	17 IU/L	7–56 IU/L
ALT	24 IU/L	10–45 IU/L

The patient was treated with recombinant factor VIII; however, there was no significant clinical improvement, prompting reconsideration of the diagnosis. This lack of response served as a critical diagnostic turning point.

Further evaluation included von Willebrand factor assays, which revealed markedly reduced ristocetin cofactor activity and reduced von Willebrand factor antigen levels, with a significantly low activity-to-antigen ratio (<0.3), indicating a qualitative defect. Ristocetin-induced platelet aggregation was also reduced. Multimer analysis demonstrated normal multimers, consistent with type 2 von Willebrand disease, particularly subtype 2M. Genetic testing identified a heterozygous missense mutation in exon 28 of the *VWF* gene, confirming the diagnosis.

Diagnosis

The final diagnosis was type 2 von Willebrand disease (likely subtype 2M), a qualitative defect in von Willebrand factor characterized by impaired platelet adhesion and reduced functional activity despite relatively preserved antigen levels. The reduced factor VIII levels observed in this patient can be explained by the role of von Willebrand factor in stabilizing factor VIII in circulation. This contributed to the initial misdiagnosis as hemophilia A.

Management and Outcome

Following confirmation of the diagnosis, treatment was modified appropriately. The patient received antifibrinolytics, hormonal therapy for menstrual regulation, desmopressin to enhance endogenous release of von Willebrand factor, and VWF-containing factor concentrates.

The patient showed significant clinical improvement with reduction in bleeding episodes and stabilization of hemoglobin levels. She was discharged with advice on long-term management, including avoidance of non-steroidal anti-inflammatory drugs and antiplatelet agents, menstrual tracking, and regular follow-up.

Comparision

These two cases highlight the diagnostic challenges in evaluating adolescents with mucocutaneous bleeding. Both patients presented with similar clinical features and normal platelet counts, yet the underlying pathophysiology differed significantly [5].

## Discussion

The evaluation of bleeding disorders requires a comprehensive understanding of hemostatic mechanisms. Glanzmann thrombasthenia results from defects in GPIIb/IIIa integrin, impairing fibrinogen binding and platelet aggregation. Patients with Glanzmann’s disease have lifelong prolonged mucosal bleeding and may require platelet transfusions for severe bleeding episodes. However, patients with repeated transfusions may become allo-immunized [5]. Some patients may have antibodies against the GpIIa/IIIa complex, which makes transfusions ineffective. These patients who are refractory to platelet transfusions can be treated with recombinant factor VIIa, which can be lifesaving or allow for future surgeries [6].

In contrast, von Willebrand disease affects platelet adhesion and factor VIII stabilization. A key diagnostic tool is platelet aggregation testing. In Glanzmann thrombasthenia, aggregation that is absent with most agonists except ristocetin should prompt further flow cytometric testing for reduced/absent αΙΙb/β3 expression and mutational analysis to identify the genetic defects of Glanzmann thrombasthenia [7]. In von Willebrand disease, ristocetin-induced aggregation is reduced, but it is limited by high coefficient of variation between results [8]. Another distinguishing feature is factor VIII levels. Reduced factor VIII is characteristic of von Willebrand disease but not Glanzmann thrombasthenia. Misdiagnosis as hemophilia can occur when factor VIII is low, emphasizing the importance of von Willebrand factor assays. A decreased von Willebrand cofactor activity by Von Willebrand antigen ratio with normal multimer distribution would lead to a diagnosis of type 2M [8].

These cases also emphasize common diagnostic pitfalls, particularly the misdiagnosis of von Willebrand disease as hemophilia in female patients with reduced factor VIII levels, which lead to confusion particularly in earlier years [9]. In the second case, the cohort and bleeding symptoms and reduced factor VIII levels initially led to a diagnosis of hemophilia A and treatment with recombinant factor VIII. The lack of clinical response prompted further evaluation, establishing the diagnosis of type 2M von Willebrand disease. Early and accurate diagnosis is crucial, as management strategies differ significantly and directly influence patient outcomes. These cases also emphasize the importance of early use of specialized platelet function and von Willebrand factor assays to avoid misclassification and ensure appropriate therapy.

Management strategies differ significantly: Glanzmann thrombasthenia requires platelet transfusion or recombinant factor VIIa, whereas von Willebrand disease is treated with desmopressin or von Willebrand factor concentrates. The mainstay of von Willebrand disease treatment is the replacement of the deficient protein [10]. These cases demonstrate the importance of integrating clinical and laboratory findings to avoid misdiagnosis and ensure appropriate treatment.

## Conclusions

These two cases highlight the diagnostic complexity of mucocutaneous bleeding disorders in adolescents, particularly when routine laboratory investigations such as platelet count and basic coagulation studies are normal or only minimally abnormal. Despite similar clinical presentations, the underlying mechanisms differed significantly, with Glanzmann thrombasthenia resulting from defective platelet aggregation due to glycoprotein IIb/IIIa dysfunction, while type 2 von Willebrand disease represented a qualitative defect in von Willebrand factor affecting platelet adhesion and factor VIII stabilization. The cases also emphasize the potential for diagnostic overlap and misclassification, particularly when reduced factor VIII levels lead to an erroneous diagnosis of hemophilia. Accurate diagnosis therefore requires careful correlation of clinical findings with targeted investigations, including platelet function analysis and von Willebrand assays, as early differentiation is essential for appropriate management and improved long-term outcomes.
